# Editorial: Understanding the Importance of Temporal Coupling of Neural Activities in Information Processing Underlying Action and Perception

**DOI:** 10.3389/fncom.2021.729296

**Published:** 2021-09-01

**Authors:** Daya Shankar Gupta, Andreas Bahmer

**Affiliations:** ^1^Biology Department, Camden County College, Blackwood, NJ, United States; ^2^Ernst Strüngmann Institut, Frankfurt, Germany; ^3^Brain Imaging Center Frankfurt, University of Frankfurt, Frankfurt, Germany

**Keywords:** binding, entropy, decorrelation, mutual information, affordance

## Introduction

The aim of this study was to understand the role of the temporal coupling of neural events in information processing underlying action and perception. Action and perception are optimized for a successful interaction of an organism with its environment to carry out tasks needed for its survival.

Information processing in the brain during an interaction with the external environment leads to an increase in mutual information and surprisal information (Gupta and Bahmer, 2019). Mutual information is a general measure of the strength of the association between two variables (Gupta and Bahmer, 2019). Pairs of variables, underlying the changes in the mutual information in the brain, may be represented by the spiking activities of pairs of nodes in a brain network, i.e., low-frequency vs. high-frequency neural oscillations during cross-frequency interaction or spiking vs. local field potential (LFP). Task-induced association between these variables due to temporal coupling can increase the mutual information and reduce the surprisal information in the brain that results from the sensory processing of stimuli, leading to a successful interaction with the external environment. Previous experimental studies have also supported an important role of temporal coupling in different perceptual tasks (Bahmer and Gupta, 2018). Moreover, the temporal coupling of neural events during information processing underlying action and perception can be of different degrees, from a less tight to a more tight degree (Gupta et al., 2020). Furthermore, binding between two brain areas or a brain activity and an external stimulus feature can emerge from temporal coupling (von der Malsburg, 1995).

Many studies in the Frontiers' Research Topics shed light on the role of the temporal coupling in information processing in the brain. Several lines of evidence presented in many contributions indicate that there is temporal coupling and an increase in mutual information during information processing in the central nervous system.

Furthermore, various parameters to study the association between neural activities, reflecting mutual information, are reported by the contributing manuscripts, which include spike–gamma LFP coherence, paired phase consistency (PPC), spike train distance, and bicoherence. In addition, many studies have highlighted the importance of synchronization in information transfer from one region of the brain to the other. Neural oscillations of slow frequency may represent the synchronization of brain networks involved in information transfer whereas high-frequency oscillations may predominantly reflect local neuronal activities (Herrmann et al., 2016). Information transfer would occur if the phase of the excitability of neurons matches with the arrival of inputs (Fries, 2015). Note that evidence from experiments based on LFP (MEG, EEG, fMRT) must be cautiously interpreted because synaptic activity from the sender projecting to the receiver network can result in false-positive coherence measures (Schneider et al., 2020). Moreover, synchronous states increase the probability of joint activity of pairs of neurons in networks, which contributes to the increase in mutual information. The synchronous activity of specific pairs of neurons, in neuron populations, modulated by tasks also reduces the surprise associated with the sensory stimuli, which contributes to a successful interaction of the brain with the environment *via* optimal perception and action (Gupta and Bahmer, 2019).

## Contributions

### Interaction With the Environment

Wen et al. investigated how task goals modulate whole brain functional connectivity when human subjects were asked to classify either taxonomic type or behavior type of behaving animals under naturalistic conditions shown in video clips. To study whole brain functional connectivity, the authors used inter-subject functional correlation. This method eliminates intrinsic signals by calculating the inter-regional correlations between different subjects performing the same task. Their findings show that whole brain functional connectivity was modulated by different task goals.

In a study of interpersonal interaction, a piano duet task was designed with three types of performer roles in the duet, namely, starting vs. joining, musical task similarity, and performer animacy (human vs. computer) (Washburn et al.). The authors noted that there are lasting effects of musical ensemble performance on attentiveness, perceptual-motor coordination, and empathy.

In a method article, Sihn and Kim studied a measure of synchrony and temporal similarity between spike trains called spike train distance. They used a method called Earth Mover's Distance (EMD) to compute spike train distance. EMD is sensitive to the temporal pattern but robust to firing rate changes. Since many of the cognitive functions are dependent on temporal patterns (Bahmer and Gupta, 2018) rather than firing rate, EMD may be used to study the effect of similarity or dissimilarity of spike patterns of pairs of neurons on cognitive functions of the brain. Smaller values of EMD in spiking of neurons suggest a greater similarity. Using the actual data from recording in the monkey motor cortex, the authors found that EMD increased as the angle was becoming orthogonal to the preferred direction. This finding underscores the importance of the temporal pattern of neuron firing in coding directional sensitivity.

In a species of weakly electric fishes (*Apteronotus leptorhynchus*), Metzen et al. investigated the coding of natural electrocommunication signals, called chirps. *A. leptorhynchus* are known to robustly give chirp echo responses when stimulated with chirps, which is used for the study of perception in these animals. Using the characteristics of chirp stimuli that are common during the interaction between same-sex conspecifics, the authors showed that synchrony in neuronal activities at all levels increased transiently in a similar fashion in response to these chirps. Furthermore, synchrony at the population level was a much better detector of the chirp stimulus than the single afferent activity. The increase in population synchrony, which promotes information transfer, was invariant to chirp attributes, namely, duration and amplitude (i.e., transient increase in frequency). The behavioral response to chirps was studied by chirp echo response rates, which was also invariant to variations in chirp attributes. During natural interactions between same-sex conspecifics, a simple behavior, giving a chirp echo response, which excludes complex behaviors, such as mating and escape, is sufficient. Thus, the invariance to chirp attributes in current conditions will not be detrimental but would only enhance their interactions with the same-sex conspecifics.

The interaction with the physical world depends on various functions of the nervous system. These include the proprioceptive performance and the discrimination of two sensory stimuli and separate movements by the shortest intervals. Odorfer et al. studied the confounds of aging and polyneuropathy on these functions by studying somatosensory temporal discrimination threshold (STDT), temporal discrimination movement threshold (TDMT), and behavioral measures of proprioception of upper and lower limbs. This study shows that aging resulted in higher STDT and TDMT but had no influence on proprioceptive performance. However, polyneuropathy resulted in higher STDT and TDMT with poor proprioceptive performance. These findings provide the objective basis for the decline in cognitive functions in older individuals, requiring interaction with their environment, such as sports activities.

### Attentional Modulation

Earlier studies showed that the default mode network (DMN) shows higher activity at rest compared with tasks involving attention. However, recent studies have suggested that DMN is also engaged during tasks involving attentional modulation. Consistent with the recent trend, Zhou et al. showed that there is a greater connectivity between two nodes of DMN, namely, posterior cingulate cortex and left inferior parietal cortex/angular gyrus in attentional tasks involving external focus in comparison with internal focus. The task paradigm required maintaining a pressure of 20 cm of water between the right index finger and thumb. External focus recruited attentional process based on the direct feedback about the pressure levels. Internal focus involved attentional modulation based on tactile sensory input and memory.

Capacity for sustained attention is important for the interaction of the brain with the environment. Wang et al. studied variability in reaction time and trial-by-trial frontal theta activity in individuals performing sustained attention tasks to understand the electrophysiological underpinnings of attention. Variability in reaction time and trial-by-trial frontal theta activity were assessed by SD and the amplitude of low-frequency fluctuation (ALFF). The authors reported that the ALFF of reaction time variability has a significant correlation with the ALFF of the trial-by-trial frontal theta activity in a frequency-dependent manner.

### Temporal Coupling

In a study by Geoly and Greene, when two complementary subsets of sparse dots are presented to represent a shape, separated by an interval between 0 and 500 ms, the probability of a correct match with the target shape remained above chance. When the complementary subsets of dots were presented simultaneously, the probability of match recognition was the highest. The probability of match recognition decreased with increasing the interval between complementary subsets of dots. These results suggest the importance of the temporal coupling of information from two subsets of dot patterns, where data from two subsets of dots are separated by the duration, ranging from 0 to 500 ms (Gupta et al., 2020). Another experiment displayed the complementary subsets with 200 ms of separation, and the match recognition was disrupted when the random dot mask was displayed midway between the two subsets. When the random dot pattern mask was presented midway between the two subsets of complementary information, it affected both subsets of information to the same extent by temporal coupling, which resulted in a complete disruption of the match recognition.

Li et al. studied spike–gamma LFP coherence by placing chronically implanted multielectrode arrays over V1 and V4 in two macaques performing a selective visual attention task. The visual stimulus used was sinusoidal gratings with orientation, between 0° and 360°, presented in pseudorandom order. The authors found that the spike–LFP synchronization strength between V1 and V4 shows orientation selectivity to drifting gratings. Li et al. further argued that synchronization between different regions not only reflects the basic features of visual stimulation but also describes the orientation tuning characteristics of neurons. This is consistent with the argument that the increase in mutual information, which results from the synchronization between different regions of the brain, can represent complex stimulus characteristics, such as orientation (Gupta and Bahmer, 2019).

van der Velden, Vinck, Werkman et al. simultaneously recorded spontaneous extracellular spikes from 10 to 30 dopamine neurons in acute slices from the lateral ventral tegmental area (VTA) of the rat. The functional connectivity between pairs of neurons was analyzed by PPC, which estimates the square of phase-locking value. Manipulating excitability with high extracellular potassium reduced the PPC, but the application of glutamate did not have any effect on PPC. Since the application of glutamate would affect excitability *via* synaptic connections, the reduced PPC after the application of high K+ is partly due to the uncorrelated activity of pairs of neurons that have no synaptic connections.

It is currently known that during speech comprehension, there is an alignment of the neuronal excitability phase of slow oscillations in the auditory cortex with slow energy fluctuations in the speech or the attributes of the speech envelope (Assaneo et al.), which is referred to as speech tracking. This alignment, indicating temporal coupling, was estimated by computing the phase-locking value between the brain activity and the cochlear envelope (Ding et al., 2017) of the perceived stream of syllables. Assaneo et al. showed that the asymmetry of the auditory tracking is reversed by the presence of semantic information even though the acoustic properties of the stimuli are similar. Hence, their findings reveal the importance of temporal binding between the auditory stimulus and the brain activity in speech perception.

Khamechian and Daliri analyzed non-linear neuronal synchronization in LFPs recorded from the middle temporal lobe signals during a visuomotor task by employing the bicoherence method to examine how non-linear neuronal synchronization in the MT area is involved in the processing of visuomotor information. Bicoherence, a study of two frequencies in a single signal, gives a maximum value if there is a perfect phase-locking and a minimum value if there is a random overlap between the phases of two frequencies. Notably, information transfer depends on phase-locking. Thus, the study of the characteristics of non-linear neuronal synchronization would be important for understanding the complex dynamics of information transfer in cognitive tasks.

In a study employing midbrain slices, van der Velden, Vinck and Wadman. combined the optogenetic stimulation of dopaminergic neurons in the VTA with the recording of action potentials. After stimulation with regular optogenetic pulses, the authors found the highest resonance at 2.9 Hz, which is the intrinsic frequency of the VTA neurons. However, after stimulation with stochastically distributed pulses, maximum resonance was noted at a subharmonic frequency of 1.5 Hz. As authors have noted, wide-band stochastic inputs to dopaminergic neurons can induce synchronous states. Also, it is noted that the synchronous states promote information flow. This is particularly interesting in the study of the response of the VTA in unpredicted rewards (Morales and Margolis, 2017). The stochastic inputs to dopaminergic neurons, resulting from unexpected rewards, can also induce synchronous states for information processing.

### Information Processing in the Brain

Using a biologically plausible simulation study, Löffler and Gupta showed that input patterns can be encoded by the coincidence detection in dendrites. When 100 Hz synchronized inputs, from I-neuron (source of activity pattern to be encoded) and A-neuron with 100 Hz regular discharge rate, coincide with the peak of the 8.33 Hz subthreshold membrane potential oscillations at one of the dendritic branches, this results in a dendritic spike leading to a somatic spike. In this model, a single dendritic spike increases the synaptic weight by ~37% at corresponding synapses. An increase in the synaptic weight at specific synapses is responsible for reproducing the same pattern of activity alone by a 100 Hz regular input even in the absence of I-neuron activity. Since synaptic weights are increased at specific synapses, depending on the temporal pattern of the input from I-neuron, this may provide a biologically plausible basis for the temporal processing of information.

In an fMRI study of dynamic functional network connectivity in subjects performing auditory discrimination and working memory tasks, the authors used the independent component analysis to extract networks, which included the auditory, the visual, the sensorimotor, the cerebellar, the frontoparietal, the default mode, and the salience networks (Zhang et al.). The sliding window analysis, with each step of 30 s and a total of 178 steps, was used to identify four states, resulting from various configurations of seven networks. The analysis of four states in two tasks revealed distinct dynamic functional connectivity of the networks. It should be noted that there are changes in mutual and surprisal information, which are due to distinctive dynamic connectivity in two tasks. This suggests that distinct dynamic connectivity rather than quantitative changes in mutual and surprisal information underlies the differences in the task goals.

A human behavioral study by Liang et al. found that the memory load, i.e., remembering an increasing visually presented list of alphanumerical items, reduces sweet and bitter taste sensitivities, which are consistent with other studies of the effects of memory load on perception.

Han and Dimitrijevic studied (1) the effect of amplitude modulation (AM) depth on the detection of amplitude modulated white noise and (2) the interaction between cortical N1 responses, hearing performance, and AM changes (4, 40, 100, and 300 Hz) in postlingually deafened subjects with bilateral cochlear implants (CIs) for speech perception in CI users. They showed that AM change stimuli can elicit robust N1 acoustic change complex responses (4 and 40 Hz) in CI users. The N1 latency to 40 Hz (not 4 Hz despite robustness) relates to speech perception measures, and temporal modulation transfer function relates well to speech perception in CI users.

In a dynamic three-node network model studied by Chen and Padmanabhan, consisting of local excitatory mitral/tufted cells and inhibitory granule cells in the olfactory bulb and excitatory cells in the piriform cortex with feedback connections to the granule cells, different computations are possible with the changes in the weight of top-down connections to the granule cells. Different computations produced by the changes in the weight of top-down connections include (1) separating two external (olfactory) stimuli based on rate coding and (2) synchronizing the oscillatory activities of two separate stimuli. The authors also suggested that weight changes can be biologically initiated by neuromodulators. Notably, while synchronization can increase the mutual information, the pattern separation based on rate coding would increase the surprisal information.

Tal et al. have reviewed the study supporting that neural oscillation may occur as transient burst-like events in addition to a sustained increase in power. Burst-like oscillations are transient events, which are separated by temporal gaps. Due to averaging over many trials to increase the signal-to-noise ratio, it was difficult to detect burst-like oscillations previous studies. However, the authors argued that the trial-by-trial changes in oscillatory dynamics must be studied to understand their role in perception and action. The authors also offered advice about choosing suitable analyses to study neural rhythms in single trials.

## Summary

A review of various contributions in the Frontiers' Research Topics suggests that synchronous activities can have the following consequences: information flow, temporal coupling, and an increase in mutual information. The synchronization would also reduce the surprise by increasing the coactivation of pairs of neurons. Notably, an initial increase in surprisal information would result from information flow. Both the increase in the mutual information and surprisal information are the important underpinnings of action and perception, subserving the interaction of the brain with the environment (Gupta and Bahmer, 2019).

## Author Contributions

All authors listed have made a substantial, direct and intellectual contribution to the work, and approved it for publication.

## Conflict of Interest

The authors declare that the research was conducted in the absence of any commercial or financial relationships that could be construed as a potential conflict of interest.

## Publisher's Note

All claims expressed in this article are solely those of the authors and do not necessarily represent those of their affiliated organizations, or those of the publisher, the editors and the reviewers. Any product that may be evaluated in this article, or claim that may be made by its manufacturer, is not guaranteed or endorsed by the publisher.
